# The ω-3 polyunsaturated fatty acids prevented colitis-associated carcinogenesis through blocking dissociation of β-catenin complex, inhibiting COX-2 through repressing NF-κB, and inducing 15-prostaglandin dehydrogenase

**DOI:** 10.18632/oncotarget.11544

**Published:** 2016-08-23

**Authors:** Young-Min Han, Migyeung Jeong, Jong-Min Park, Mi-Young Kim, Eun-Jin Go, Ji Young Cha, Kyung Jo Kim, Ki Baik Hahm

**Affiliations:** ^1^ CHA Cancer Prevention Research Center, CHA Cancer Institute, CHA University, Seoul, Korea; ^2^ Gachon University Lee Gil Ya Cancer and Diabetes Institute, Incheon, Korea; ^3^ Department of Gastroenterology, University of Ulsan, Seoul Asan Hospital, Seoul, Korea; ^4^ Department of Gastroenterology, CHA Bundang Medical Center, Seongnam, Korea

**Keywords:** fat-1 transgenic mice, colitic cancer, COX-2, ω-3 PUFAs, 15-PGDH, β-catenin complex

## Abstract

Numerous studies have demonstrated that diets containing an increased ratio of ω-6 : ω-3 polyunsaturated fatty acids (PUFAs) are a risk factor for colon cancer and might affect tumorigenesis. Therefore, dietary ω-3 PUFA administration may be a preventive strategy against colon cancer. Until now, the exact molecular mechanisms and required dietary doses of ω-3 PUFAs for cancer prevention were unknown. In this study, we explored the anti-tumorigenic mechanisms of ω-3 PUFAs against a colitis-associated cancer (CAC) model. Through *in vitro* cell models involving docosahexaenoic acid (DHA) administration, down-regulation of survivin and Bcl-2, and up-regulation of Bax, accompanied by blockage of β-catenin complex dissociation, the main mechanisms responsible for DHA-induced apoptosis in HCT116 cells were determined. Results included significant reduction in azoxymethane-initiated, dextran sodium sulfate-promoted CACs, as well as significant preservation of 15-hydroxyprostaglandin dehydrogenase (15-PGDH) and significant inhibition of Cyclooxyganase-2 (COX-2) and Prostaglandin E_2_(*P* < 0.01). Additional mechanisms and significant induction of apoptosis in both tumor and non-tumor tissues were also noted in *fat*-1 transgenic (TG) mice. The lipid profiles of colon tissues measured in all specimens revealed that intake greater than 3 g ω-3 PUFA/60 kg of body weight showed tissue levels similar to those seen in *fat*-1 TG mice, preventing cancer. Our study concluded that COX-2 inhibition, 15-PGDH preservation, apoptosis induction, and blockage of β-catenin complex dissociation contributed to the anti-tumorigenesis effect of ω-3 PUFAs, and an intake higher than 3g ω-3 PUFAs/60 kg of body weight can assist in CAC prevention.

## INTRODUCTION

The *fat*-1 transgenic (TG) mice [1] and *fat*-1 TG cattle [2] were designed to convert ω-6 to ω-3 polyunsaturated fatty acids (PUFAs) through transgenic expression of n-3 desaturase enzyme and have been acknowledged as an ideal model to study the effect of endogenous ω-3 PUFAs on various diseases, from benign metabolic and inflammatory diseases to several kinds of cancer. Before the availability of these TG animals, most *in vivo* studies exploring the influence of ω-3 PUFAs involved modifying tissue nutrient composition by supplementing the experimental groups with different ω-6 : ω-3 fatty acid ratios because mammals cannot convert ω-6 to ω-3 fatty acids intrinsically because of a lack of *n*-3 desaturase [3]. Therefore, the inevitable differences between dietary intake and their real components, however small they may be, contribute to inconsistent and conflicting observations. The limitation of using fish or plant oils to provide ω-6 : ω-3 PUFAs can be overcome by developing a TG animal using a genetic approach, modifying the ω-6 : ω-3 ratio via expression of the *Caenorhabditis elegans fat*-1 gene encoding *n*-3 desaturase in mammalian cells. Results obtained in these TG models will be more reliable for interpreting the exact function of ω-3 PUFAs [4].

After the introduction of *fat-*1 TG mice by Kang *et al.* [4], numerous publications documented the cancer preventive effects of ω-3 PUFAs against diverse cancer models such as lung cancer [5], melanoma [6], pancreatic cancer [7], breast cancer [8], prostate cancer [9], liver tumor [10, 11], colorectal cancer, and colitic associated cancer (CAC) [12–14]. In spite of abundant reports consistently showing anti-tumorigenic effects of ω-3 PUFAs mediated via their anti-inflammatory, anti-oxidative, anti-proliferative, and anti-mutagenic properties, most studies simply describe the mechanisms commonly explaining anti-tumorigenesis. Specifically, there is no published paper that shows the extent to which the exogenous ω-3 PUFAs should be supplemented to achieve anti-tumorigenic effects similar to those achieved in *fat-*1 TG mice. Among the aforementioned diverse reports describing models for cancer prevention by ω-3 PUFAs, only three publications on the pancreatic ductal adenocarcinoma model containing p48^Cre/+−^LSL-Kras^G12D/+^ [7], the colon adenoma model containing APC^min/+^ [15], and the breast cancer model describing down-regulation of the transmembrane tyrosine kinase receptor HER2 pathway [8], showed specific mechanisms explaining cancer prevention by ω-3 PUFAs in *fat-*1 TG mice. The remaining reports discussed general mechanisms implicated in cancer prevention, such as anti-inflammation, anti-proliferation, and anti-oxidation.

*In vivo* study involving azoxymethane (AOM)-initiated, dextran sulfate sodium (DSS)-promoted CACs, along with reporting that *fat-*1 TG mice showed significantly lower incidence of cancer, we explored the cancer preventive mechanisms of docosahexaenoic acid (DHA), including selective cytotoxicity against colon cancer cells by apoptosis and cell cycle inhibition, blocking the dissociation of β-catenin complex, and induction of tumor suppressive 15-prostaglandin dehydrogenase (15-PGDH), beyond the reported anti-proliferative, anti-inflammatory, and anti-oxidative actions. For future clinical applications, we have added the informative finding that dietary intake of more than 3 g ω-3 PUFAs / 60 kg of body weight could achieve similar tissue levels of ω-6 : ω-3 PUFAs as seen in colon of *fat-*1 TG mice.

## RESULTS

### DHA-regulated abnormal cellular proliferation via apoptosis and blocking the disassociation of β-catenin complex in HCT116 colon cells

**Figure 1:**
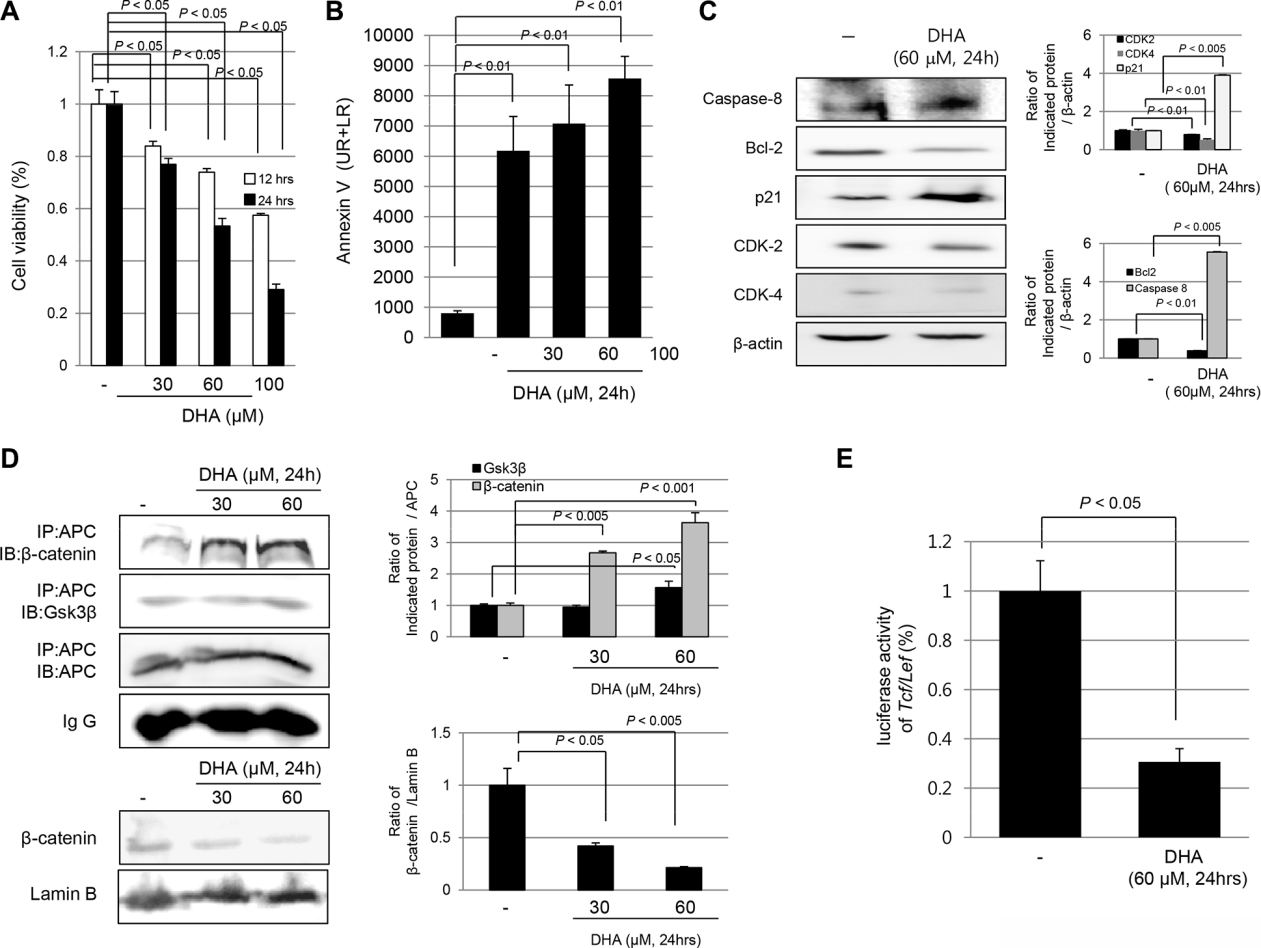
DHA induced cytotoxicity of HCT116 colon cancer cells via apoptosis and blocking the disassociation of β-catenin complex (**A**) Cell viability was measured with MTT assay. DHA significantly decreased cell viability in a time- and dose-dependent manner (*P* < 0.05). (**B**) HCT116 cells were treated with DHA (30, 60 and 100 μM/ml) for 24 hours and analyzed for FITC annexin V and propidium iodide staining by flow cytometry. The percentage of cells in apoptosis was determined by using the Mod-Fit program. (**C**) HCT116 cells were challenge with DHA for 24 h to investigate the expression of cell cycle and apoptotic markers including capspase-8, Bcl-2, p21, CDK-2, and CDK-4. The expressions of indicated antibodies were assessed by Western blot analysis. (**D**) HCT116 cells were treated with different concentrations of DHA for 24 h and cell lysates were immunoprecipitated with anti-APC antibody followed by immunoblotting with GSK3β, β-catenin, and APC antibody, respectively (upper). Western blots for expression of β-catenin in nucleus following DHA treatment (lower). (**E**) HCT116 cells were transiently transfected with the *Tcf*/*Lef*-*Luc* reporter vector. After transfection, the cells were cultured in serum-free medium with DHA for 12 h and the cell lysates were obtained to measure the luciferase activity.

To determine whether DHA induced cytotoxicity, HCT116 cells were treated with different doses of DHA (30, 60, and 100 μM/ml) for 12 and 24 h each. As seen in Figure 1A, DHA significantly induced cytotoxicity in a dose-dependent manner in HCT116 cells (*P* < 0.05). To determine whether DHA cytotoxicity could be attributed to its apoptotic action, we performed flow cytometry after staining with fluorescein isothiocyanate (FITC)-Annexin V, a biochemical marker of apoptosis. As seen in Figure 1B, DHA significantly induced apoptosis in HCT116 cells dose-dependently (*P* < 0.01). These findings from flow cytometry were further validated by Western blot analysis for caspase-8, Bcl-2, p21, CDK-2, and CDK-4 (Figure 1C). When cells were treated with 60 μM DHA, the levels of caspase-8, as apoptotic executor, and p21 were significantly increased (*P* < 0.01), but those of B-cell lymphoma 2 (Bcl 2), Cylin dependent kinase (CDK)-2, and CDK-4 were significantly decreased (*P* < 0.01), indicating that DHA induced cytotoxicity through induction of apoptosis. With the hypothesis that DHA might regulate cell proliferation through maintaining the β-catenin complex to inhibit nuclear translocation of β-catenin, we examined the effects of DHA on β-catenin transactivation status in HCT116 cells. As shown in Figure 1D, DHA treatment significantly increased either the levels of β-catenin or Glycogen synthase kinase 3β (GSK3β) with Adenomatous polyposis coli (APC) binding (*P* < 0.001), which can block the dissociation of either GSK3β or β-catenin associated with, blocking the dissociation of the β-catenin complex in a dose-dependent manner; Through increase of either β-catenin or GSK3β, DHA inhibited excess cellular proliferation under optimal cytoplasmic levels of β-catenin, blocking the dissociation of the β-catenin complex (Figure 1D). Therefore the nuclear expression of β-catenin was increased in HCT 116 cells, whereas DHA-treated cells showed that decreased expression of β-catenin in nucleus. Furthermore, DHA treatment significantly decreased *Tcf/Lef* reporter activity (*P* < 0.05, Figure 1E) to repress β-catenin-associated abnormal cellular proliferation. All of these results suggest that DHA prevented an abnormal β-catenin-associated proliferative signaling pathway via increased GSK3β-binding to APC and prevented an abnormal β-catenin destruction complex in HCT116 cells.

### DHA inhibited COX-2 and NF-κB activation and induced 15-PGDH expression

**Figure 2:**
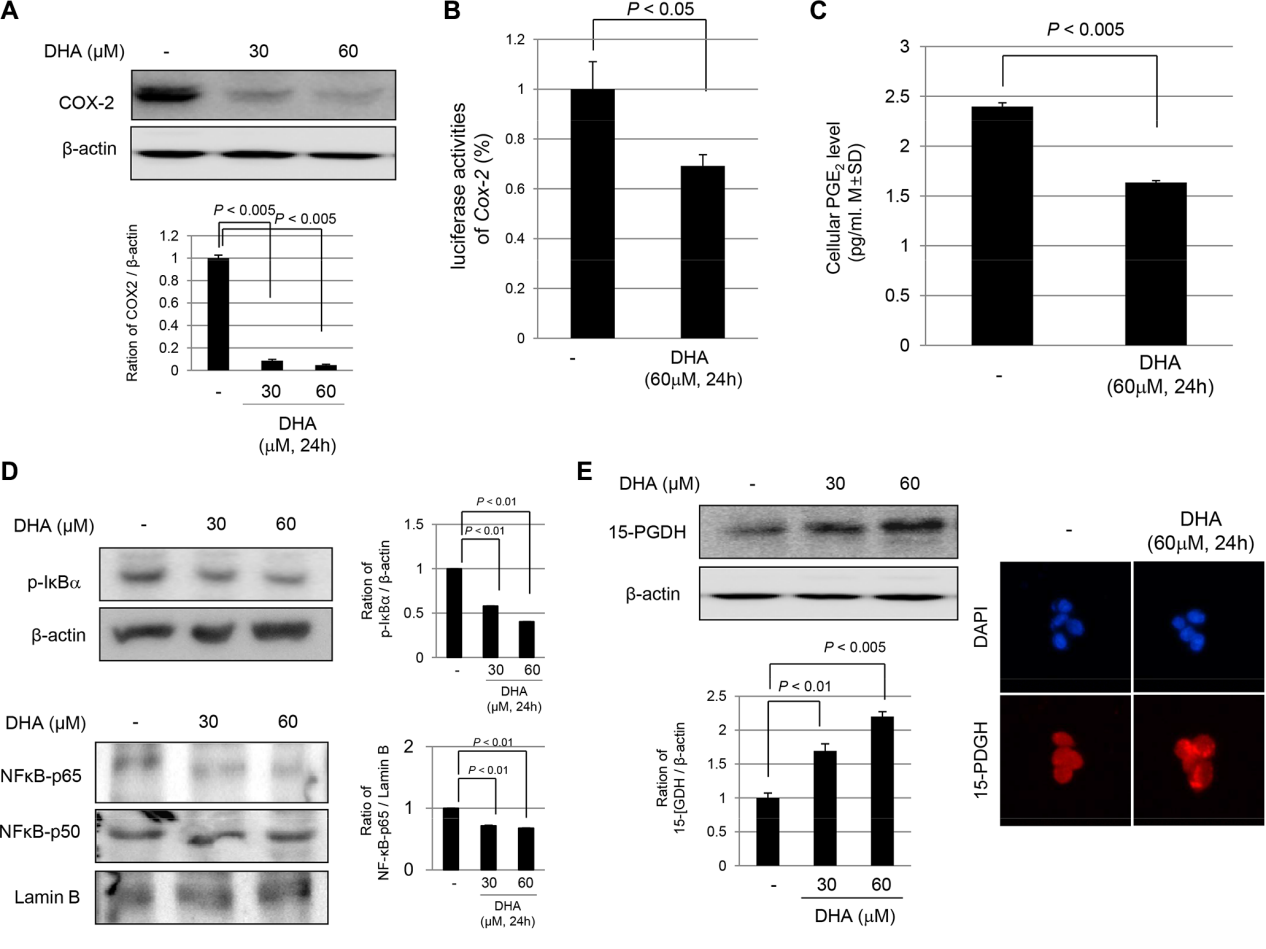
DHA inhibited COX-2, repressed NF-kB activation, but induced 15-PGDH expression (**A**) COX-2 inhibition by DHA. HCT116 cells were treated with 30 and 60 μM DHA in medium for 24 h and then, the cell lysates were obtained for Western bolt analysis using COX-2 antibody. (**B**) COX-2 promoter activity after 60 μM DHA. HCT116 cells were transiently transfected with a luciferase reporter construct controlled by the COX-2 promoter. After transfection, the cells were treated with vehicle or DHA in medium. The cell lysates were obtained to determine the luciferase reporter activity. (**C**) The changes of PGE_2_ after DHA. PGE_2_ levels were measured by ELISA. (**D**) The Western blots for cytoplasmic levels of phosphorylated IkBa and β-actin and nuclear levels of NF-κB p65, p50 and Lamin B after different dose of DHA. (**E**) The expression of 15-PGDH was investigated by either Western blot analysis (left) or confocal imaging (right, ×200) after treatment of DHA.

As another anti-tumorigenic action of Cyclooxygenase 2 (COX-2)inhibition and Nuclear factor kappa-light-chain-enhancer of activated B cell (NF-κB)inactivation are reported as additional mechanisms of the antitumorigenic action of ω-3 PUFAs. Therefore, we examined the effect of DHA on COX-2 expression. As shown in Figure 2A and 2B, DHA significantly inhibited the expression of COX-2 in a dose-dependent manner for 24 h (*P* < 0.005), and DHA directly inhibited COX-2 promoter activity (*P* < 0.05) in HCT116 cells, suggesting that DHA inhibited the expression of COX-2 in part through the suppression of gene transcription. Consequently, the levels of Prostaglandin E_2_ (PGE_2_) (a product of the COX-2 enzyme action) were significantly decreased in DHA-treated HCT116 cells (*P* < 0.005, Figure 2C). In order to check transcriptional repression by DHA, NF-κB expression was checked in either cytoplasmic homogenates or nuclear fraction. As seen in Figure 2D, DHA significantly decreased Nuclear factor of kappa light polypeptide gene enhancer in B-cells inhibitor, alpha (IκBα) phosphorylation (*P* < 0.01), and nuclear translocations of NF-κB p65 were significantly decreased (*P* < 0.01). As with changes to COX-2 by DHA, 15-PGDH has been reported to be a significant tumor-suppressive gene affecting COX. DHA treatment significantly increased the expression of 15-PGDH in a dose-dependent manner (*P* < 0.01, Figure 2E); this increase in expression was further validated by confocal imaging after staining with anti 15-PGDH antibody. Significantly increased expression of 15-PGDH in the cytoplasm of HCT116 cells was noted. These observations from the *in vitro* experiment consistently suggested that DHA could be applied to prevent inflammation-based carcinogenesis. Hence, we compared the AOM-initiated, DSS-promoted colitis-associated carcinogenesis in Wild-type (WT) and *fat-*1 TG mice to obta *in vivo* confirmation that ω-3 PUFAs can prevent CAC.

### Significantly attenuated colitis-associated cancer in *fat*-1 TG mice compared to WT mice

**Figure 3:**
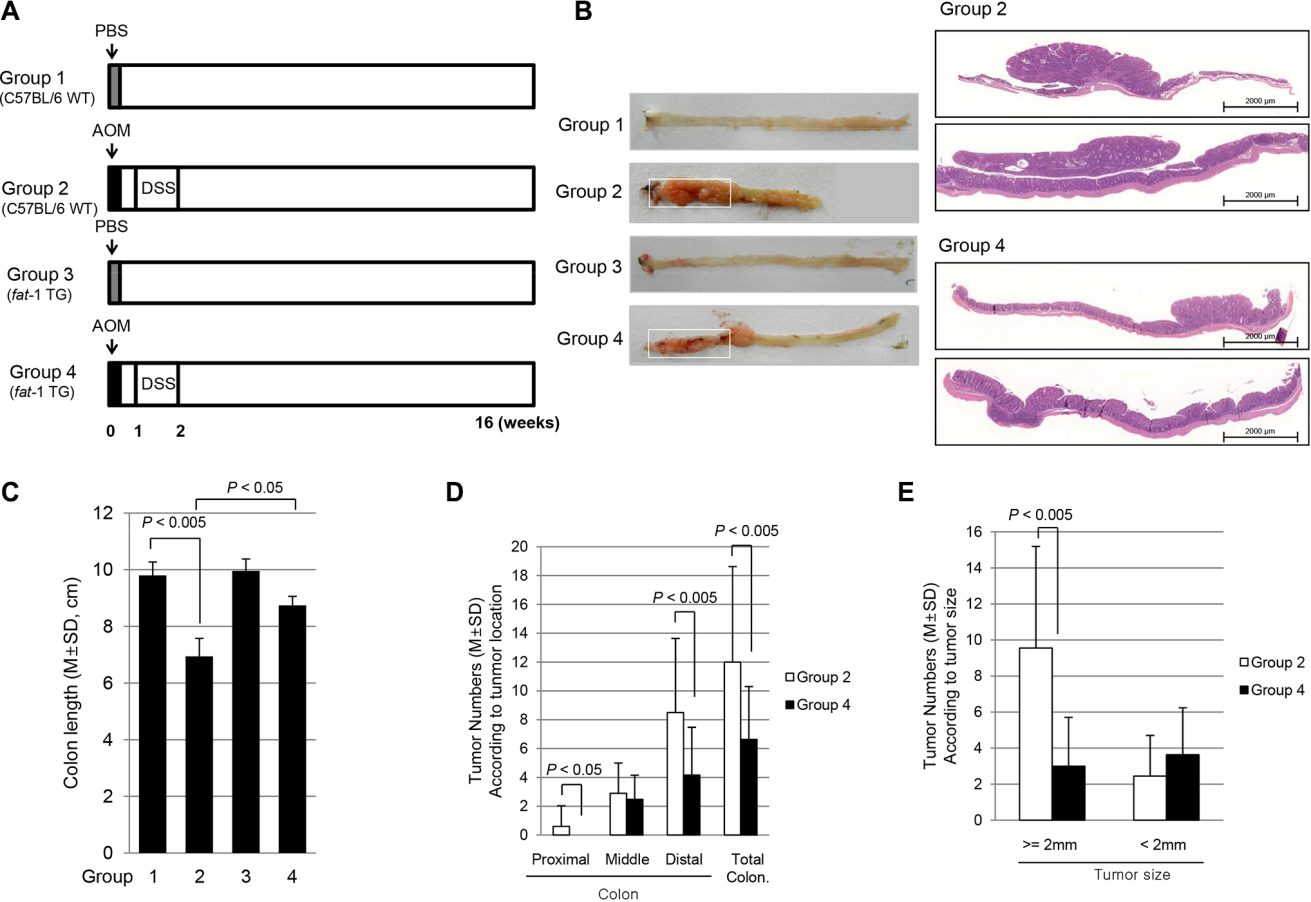
AOM-initiated, DSS promoted *in vivo* model of colitis-associated carcinogenesis and the influence of ω-3 PUFAs on colitic cancer prevention (**A**) The schematic overview of the experimental protocol for colitic cancer using C57BL/6 and *fat-*1 TG mice. The experimental animal (*n* = 8) were divided 4 groups. (**B**) Representational gross and microscopic pictures according to group. (**C**) Mean changes of colon length according to group. **(D**) Tumor numbers according to site of colon tumor. Colon tumors were counted according to proximal 1/3, mid 1/3, and distal 1/3. (**E**) Comparison of tumor number according to tumor size, < 2 mm and > 2 mm.

To assess the *in vivo* efficacy of ω-3 PUFAs in the prevention of CAC, we used C57BL/6 mice as WT and *fat-*1 TG mice for inducing AOM-initiated, DSS-promoted CAC. The carcinogen AOM is used to introduce genomic mutations by methylation, followed by repeated rounds of DSS to induce a chronic pattern of colitis [16]. As reported before, mice developed grossly visible multiple tumors starting from the rectum, extending to mid-colon (Figure 3B). As seen in Figure 3B, AOM and DSS administration provoked significant development of colorectal tumors accompanied by the significant presence of colon inflammation. On 1:1 mounted pathology, the tumors in Group 2 were significantly larger than tumors developed in Group 4. As seen in Figure 3C, the mean colon length was significantly decreased in Group 2 (*P* < 0.005) compared with Group 1, but the mean colon length of mice in Group 4 was significantly increased compared to that of Group 2 mice (*P* < 0.05), suggesting mitigated colitis in Group 4 might be responsible for decreased tumorigenesis. On analysis of tumorigenesis according to site of tumor (Figure 3D) and size of tumor (Figure 3E), significantly decreased incidence of tumorigenesis was noted in the distal colon of *fat-*1 TG mice (*P* < 0.005) and these same mice displayed smaller tumor size (*P* < 0.005). On detailed pathological analysis, all tumors arising in WT mice were moderately differentiated adenocarcinoma and some cancers invaded the passing submucosal layer, whereas tumors arising in *fat-*1 TG mice were adenoma in 70% and well-differentiated adenocarcinoma in 30%.

### Attenuated expression of COX-2, repressed NF-κB transcriptional activities, and increased 15-PGDH levels in *fat*-1 TG mice compared to WT mice

**Figure 4:**
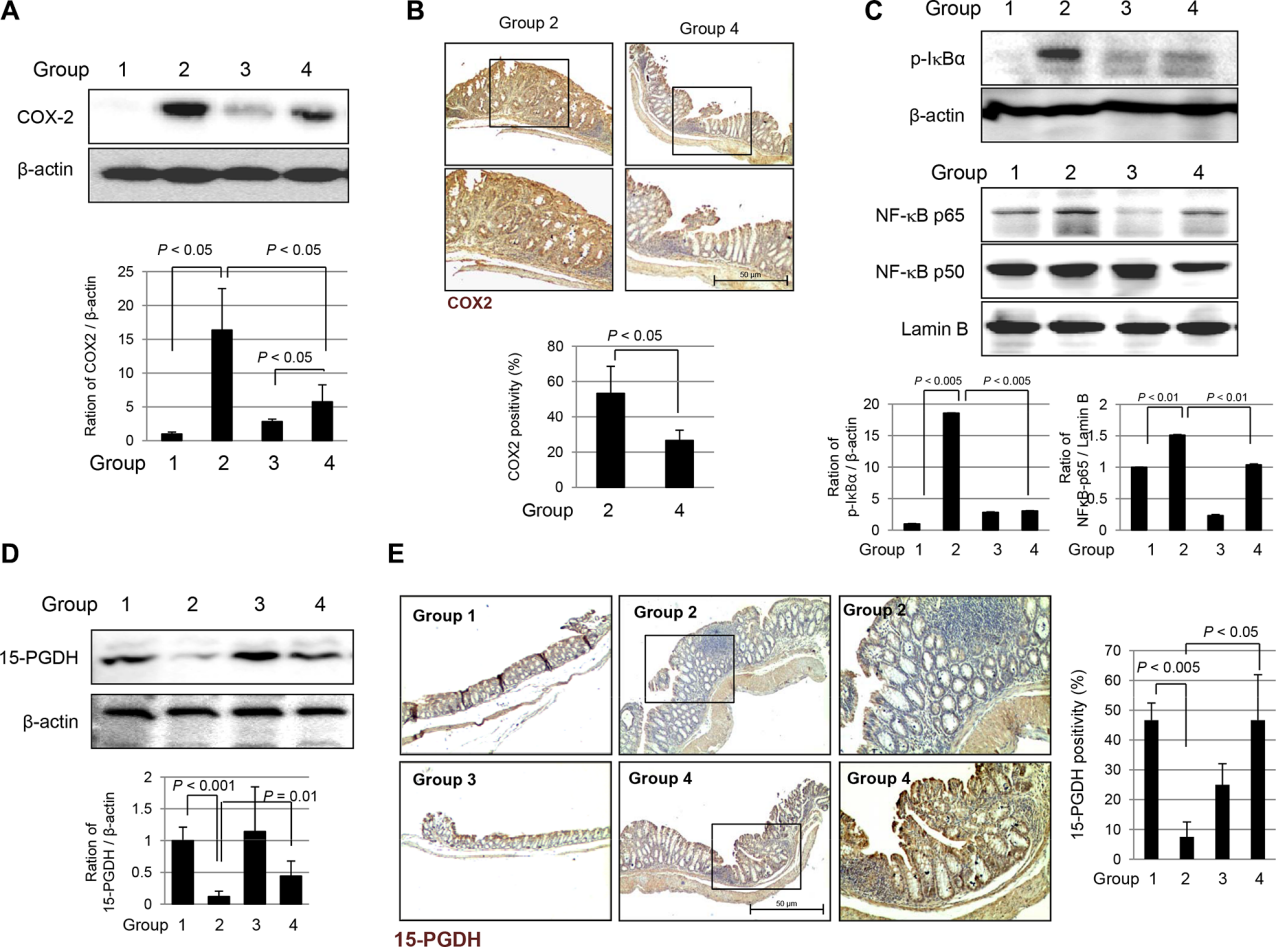
Expression changes of COX-2, NF kB and 15-PGDH according to experimental groups (**A**) Western blot for COX-2 expression. Significantly increased expressions of COX-2 in Group 2, but attenuated in Group 4. (**B**) Immunohistochemical stainings for COX-2. (Magnification at ×100, upper and magnification at × 200, lower). (**C**) Western blotting for cytoplasmic expression of phosphorylated IkBa and β-actin and nuclear expression of NF-kB p65, p50 and Lamin B according to group. (**D**) Western blot for 15-PGDH according to group. (**E**) Immunohistochemical staining of 15-PGDH according to group. (Magnification at × 100, left and middle and magnification at × 200, right).

AOM-initiated, DSS-promoted colitis-associated carcinogenesis is featured with increased COX-2 and NF-κB redox-sensitive transcriptional activation. As seen in Figure 4A, significantly increased expression of COX-2 was noted in Group 2, but these levels were significantly decreased in Group 4 (*P* < 0.05). On immunohistochemical staining with COX-2 antibody to compare the expression of COX-2 according to group, there was significant difference in COX-2 expression between WT mice and *fat-*1 TG mice (*P* < 0.05, Figure 4B). Significantly increased expression of COX-2 was noted in both the epithelia of colon crypt and infiltrated inflammatory cells in the lamina propria. Since NF-κB is an important transcription factor mediating pro-inflammatory signaling transcriptional activation of various inflammatory genes [17], we performed Western blots in cytoplasmic and nuclear fractions on homogenates of each group. As seen in Figure 4C, phosphorylated IκBα was significantly increased in Group 2 (*P* < 0.005), which can lead to increased nuclear translocation of NF-κB p65 (*P* < 0.01). However, phosphorylated IκBα and NF-κB p65 were significantly attenuated in Group 4, suggesting ω-3 PUFAs significantly repressed NF-κB transcriptional activation. Mucosal levels of PGE_2_ were significantly decreased in Group 4 compared to levels in Group 2 (*P* < 0.01, data not shown). 15-PGDH has been acknowledged as an important tumor suppressive gene, especially in preventing colitis-associated cancer. As seen in Figure 4D, significantly decreased levels of 15-PGDH were noted in Group 2 compared to those in Group 1 (*P* < 0.001), signifying that AOM-initiated, DSS-promoted carcinogenesis is associated with cancellation of 15-PGDH. However, the expression of 15-PGDH was significantly preserved in Group 4 (*P* < 0.01). On repeated validation of 15-PGDH expression with immunohistochemical staining, as seen in Figure 4E, significantly decreased levels of 15-PGDH were observed in Group 2, while significantly preserved expression of 15-PGDH was observed in Group 4 (*P* < 0.05).

### Significantly decreased Ki-67, Cyclins and inactivation of β-catenin were noted in *fat*-1 TG mice compared to WT mice during AOM-initiated, DSS-promoted colon carcinogenesis

**Figure 5:**
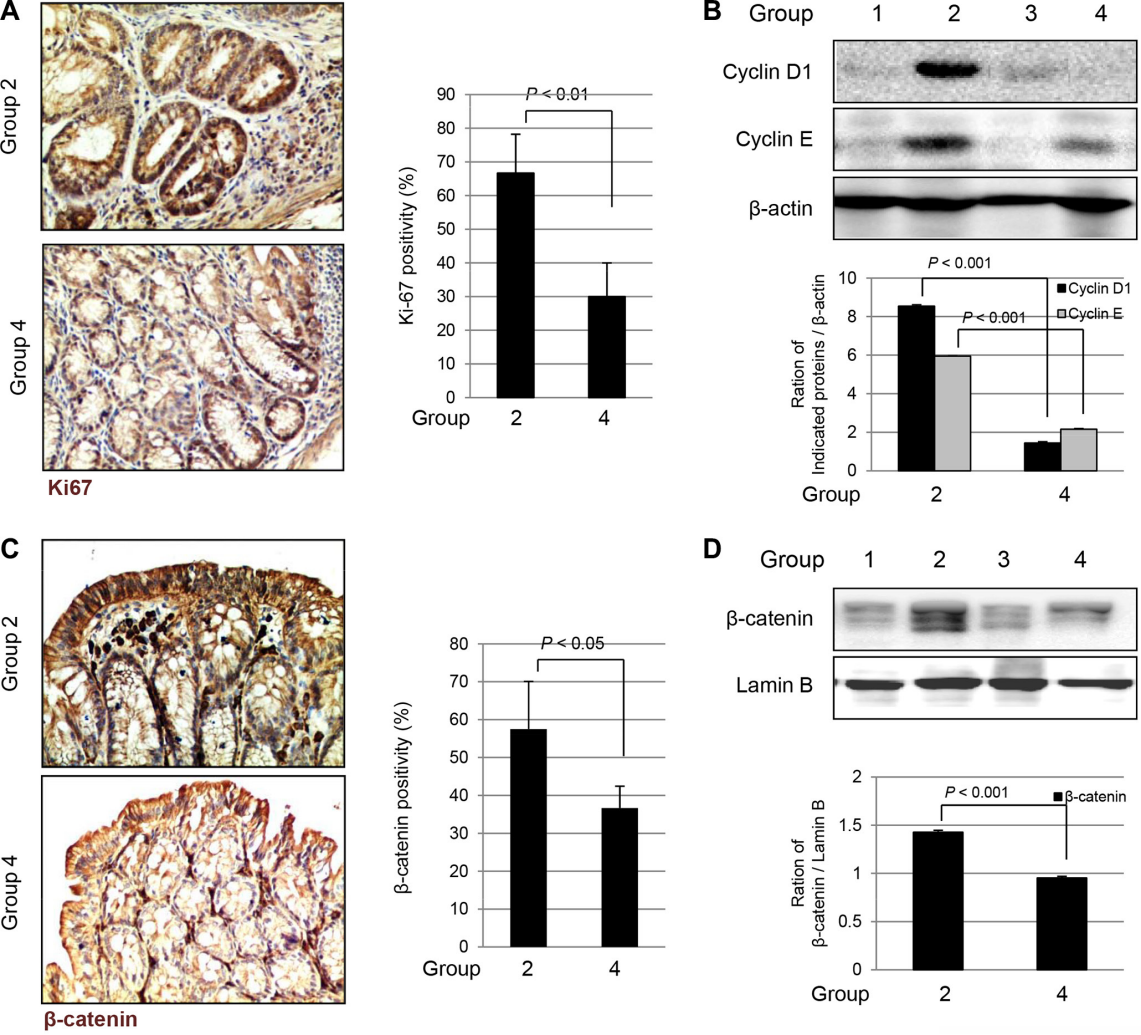
The changes of Ki-67, Cyclins, and β-catenin according to group (A) Immunohistochemical staining of Ki67 (Magnification at × 100) (**B**) Western blots for Cyclin D1 and Cyclin E according to group. The expressions of Cyclin D1 and Cyclin E were significantly decreased in Group 4 compared to Group 2 (*P* < 0.01). (**C**) Immunohistochemical staining of β-catenin, magnification at ×100. (**D**) Western blots for expression of β-catenin in nucleus of tissue homogenates. The expression of β-catenin in nucleus was significantly decreased in Group 4 compared to Group 2 (*P* < 0.001).

Since the tumor cells show high cellular proliferation, we measured the expressions of Ki-67 and cyclins. As seen in Figure 5A, significantly increased expression of Ki-67 was noted by immunohistochemical staining with anti-Ki-67 antibody in Group 2; increased expression of Ki-67 was seen in the cytoplasm and nucleus of colon crypts in tumorous areas as well as in surrounding non-tumorous areas. However, Ki-67 expression was significantly decreased in Group 4 (*P* < 0.01). In addition, we checked the cell cycle associated factors such as cyclin D1 and cyclin E with Western blot. The expressions of cyclin D1 and cyclin E were significantly increased in Group 2, but not in Group 4 (*P* < 0.001, Figure 5B). In order to document how *fat-1* TG mice inhibited the colitis-associated cancer induced by combination of AOM and DSS, we hypothesized that the anti-tumorigenic effect of *fat-*1 TG mice might be due to subsequently decreased nuclear translocation of β-catenin. As seen in Figure 5C, β-catenin was increasingly expressed in the colonocyte nucleus, whereas Group 4 showed staining of β-catenin in the colonocyte membrane. β-catenin expression was increasingly noted in the colonocyte nuclei of Group 2; however, in Group 4, its expression was not strong and stained primarily the colonocyte membranes (*P* < 0.001), suggesting blocking of β-catenin nuclear translocation occurred in Group 4. These findings were further validated with western blotting for nuclear β-catenin expressions according to group. As seen in Figure 5D, the nuclear expression of β-catenin was significantly increased in Group 2, whereas its expression in Group 4 was significantly changed compared to those seen in Group 2 (*P* < 0.001), suggesting that inhibition of β-catenin translocation from cytoplasm to nucleus might explain the anti-cancer effects in *fat*-1 TG mice, similar to findings in *in vitro* documentation that DHA significantly regulated β-catenin activation (Figure 1D).

### Increased apoptosis and apoptotic executors in *fat*-1 TG mice

**Figure 6:**
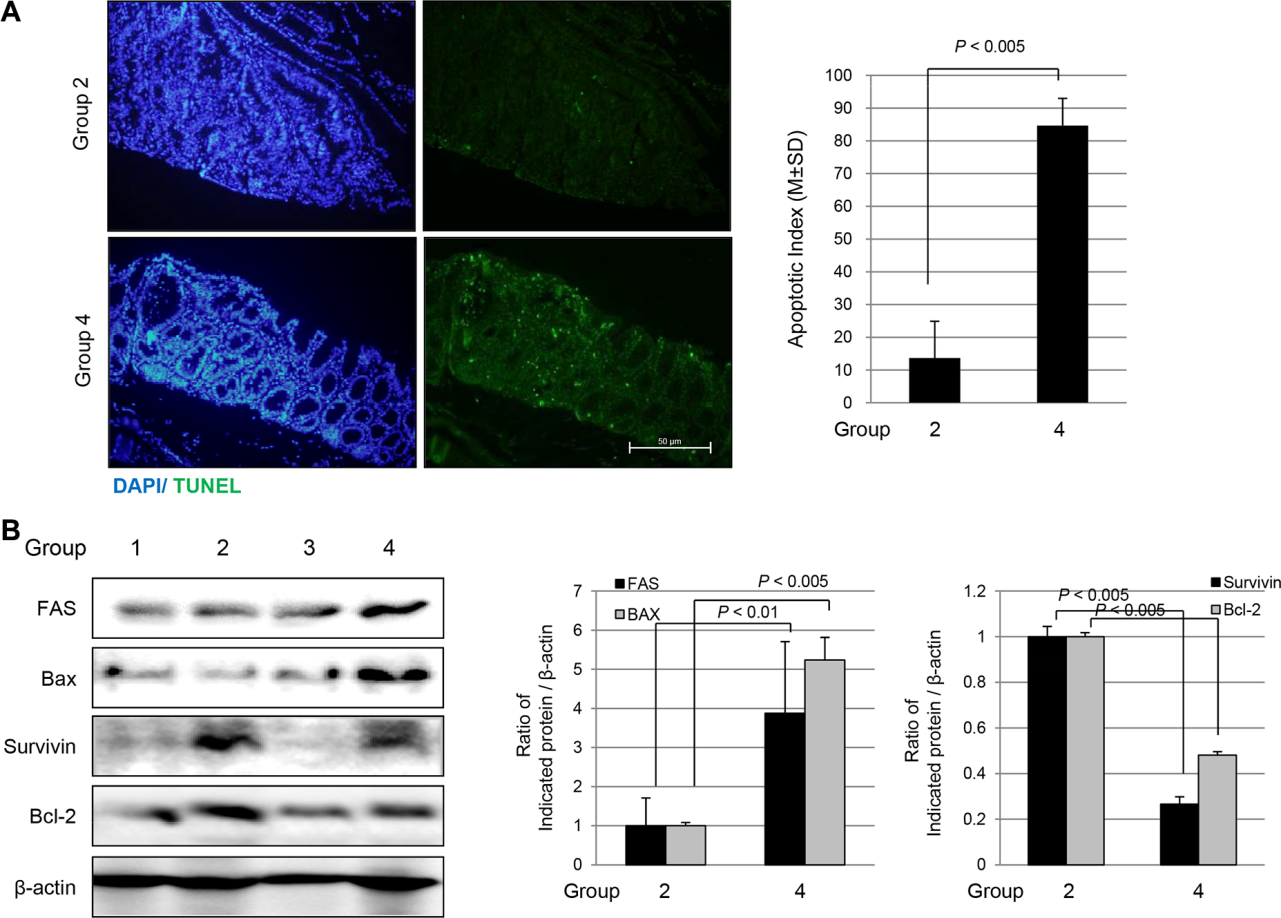
Apoptosis assessed with TUNEL staining and Western blots according to group (**A**) TUNEL staining to measure apoptotic index according to group (magnification at × 100). (**B**) Western blots for FAS and Bax as apoptotic executor and survivin and Bcl-2 as anti-apoptotic molecules according to group.

Supported by *in vitro* experiment showing ω-3 PUFAs afforded cytotoxicity in cancer cells (Figure 1), we have performed a Terminal deoxynucleotidyl transferase dUTP nick end labeling (TUNEL) assay to compare the apoptotic activities according to group. As seen in Figure 6A, a significantly increased apoptotic index assessed by assay was noted in Group 4 compared to that found in Group 2 (*P* < 0.005). Significantly increased TUNEL positivity was noted in both the tumor and non-tumor areas of Group 4. Western blot analysis was used to check the expressions of apoptotic molecules including FAS and Bcl-2-associated X protein (Bax), and the expressions of anti-apoptotic molecules including survivin and Bcl-2. As seen in Figure 6B, significantly increased expressions of FAS and Bax were observed in Group 4 compared to expressions observed in Group 2 (*P* < 0.01), while the expression of Bcl-2 and survivin was significantly decreased in Group 4 compared to that in Group 2 (*P* < 0.005).

### Similar colon lipid profiles of *fat*-1 TG mice can be achieved in WT mice administered with exogenous 3 g ω-3 PUFAs /60 kg of body weight

**Figure 7:**
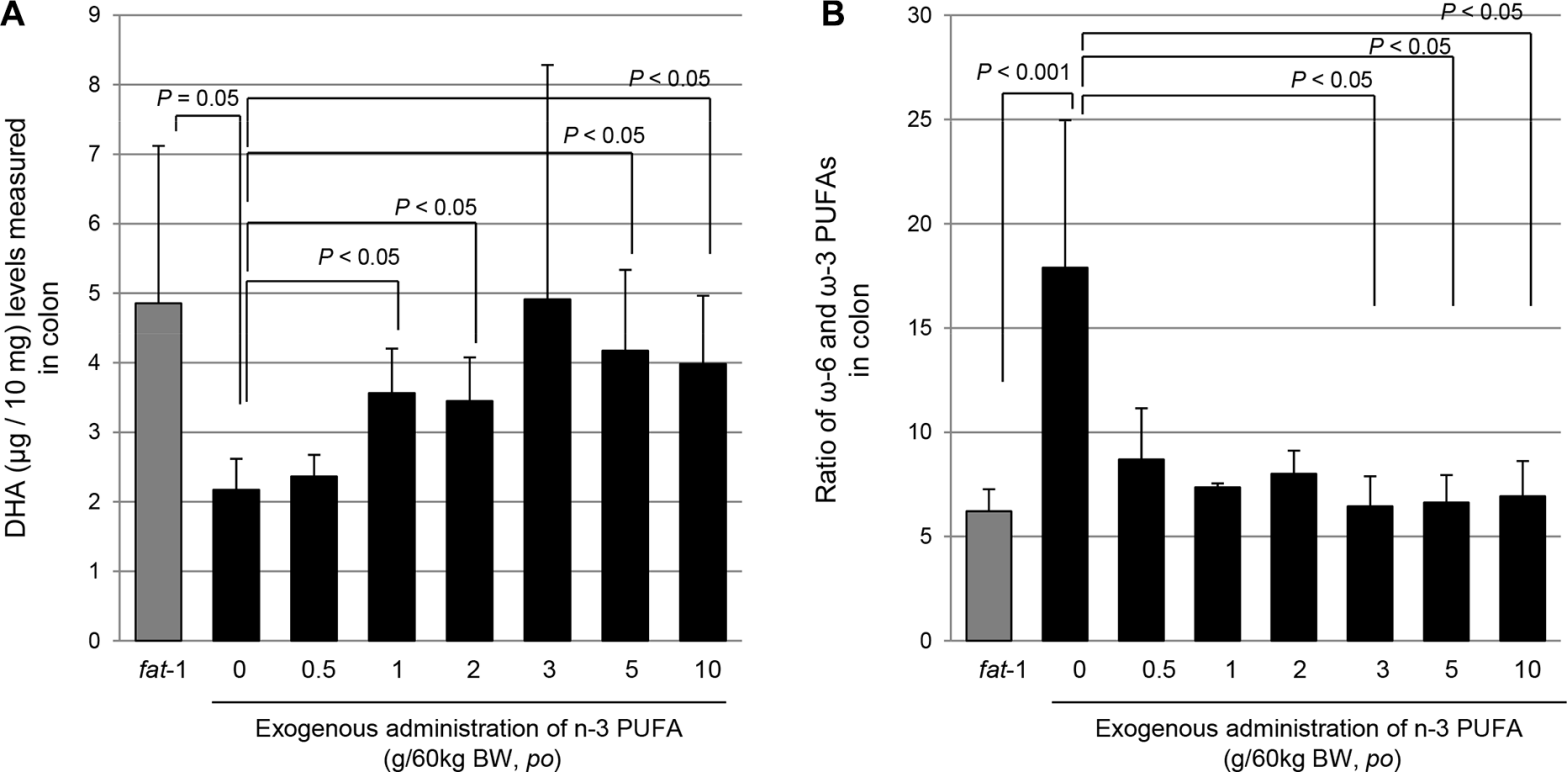
Measurement of colon levels of DHA in *fat*-1 TG mice and WT mice administered with different dose of ω-3 PUFAs with GC/MS/MS (**A**) Gas chromatography-Mass spectroscopy for measuring ω-6 and ω-3 PUFA from colon homogenates, left showing lipid profile from WT mice and right lipid profile from *fat*-1 TG mice. (**B**) The colon levels of DHA were measured in *fat-*1 TG mice fed with usual diet and WT mice fed with diet containing 0.5, 1, 2, 3, 5, and 10 g ω-3 PUFAs/60 kg of body weight. (**C**) Calculation of ω-6 : ω-3 PUFAs ratio in *fat-*1 TG mice and WT mice fed with different dose of ω-3 PUFAs. Lipid profiles were analyzed by LC/MS/MS as described in Materials and Methods.

To determine how much exogenous ω-3 PUFAs is required to translate the current lipid profiles shown in *fat-*1 TG mice to future clinical applications, we administered exogenous ω-3 PUFAs to WT mice and measured colon lipid profiles in colon tissues of *fat-*1 TG mice and WT mice administered with 0.5, 1, 2, 3, 5, and 10 g ω-3 PUFAs / 60 kg of bodyweight *po*, respectively through GC-MS/MS. As seen in Figure 7, the specimens were divided into seven groups: a *fat*-1 TG group and six groups of C57BL/6 WT mice receiving exogenous ω-3 PUFAs of 0.5, 1, 2, 3, 5 and 10 g ω-3 PUFAs / 60 kg of bodyweight, respectively. We gave exogenous ω-3 PUFAs to WT mice through oral gavage for one week and sacrificed all mice; GC-MS/MS analysis using resected colon tissue followed. As seen in Figure 7A, spectroscopic peaks for ω-3 PUFA denoting α-linolenic acid (ALA), Eicosapentaenoic acid (EPA), and DHA, were noted only in *fat*-1 TG mice, while only AA spectra were seen in WT mice. When we compared DHA levels in the colon, *fat*-1 TG mice contained 4.8 μg of DHA in 10 mg colon tissue, significantly higher than that found in WT mice (*P* < 0.05). Though administration of more than 1 g exogenous ω-3 PUFAs / 60 kg of body weight significantly increased tissue levels of DHA (*P* < 0.05), mice administered with more than 3 g ω-3 PUFAs / 60 kg of body weight, showed a mean of 4.9 μg DHA in 10 mg colon tissue, equivalent to levels seen in the *fat*-1 TG mice group (*P* < 0.05, Figure 7B). Calculating lipid profiles as a ratio of ω-6 to ω-3 PUFAs, since the ratio of ω-6 to ω-3 PUFAs remains 10–4:1 in humans other than those with a Western diet and 20–15:1 in the case of Western diet deficient in ω-3 PUFAs [18], as seen in Figure 7C, *fat-*1 TG mice showed a significantly lower ratio of ω-6 : ω-3 PUFAs (*P* < 0.001) and intake of more than 3 g ω-3 PUFAs / 60 kg of body weight showed a ratio similar to that seen in *fat-1* TG mice (*P* < 0.05). The *fat-*1 TG mice group analysis indicated the optimal balance of ω-6 to ω-3 PUFAs was 6:1, whereas the WT mice group analysis demonstrated a ratio of 17:1. Exogenous intake of ω-3 PUFAs, DHA in the current study, led to an optimal ratio of 8–6:1 in colon tissue. Considering these results, we hypothesized that intake of more than 3 g ω-3 PUFAs / 60 kg of body weight can provide similar preventive effects in colitis-associated cancer, even though we did not confirm anti-tumorigenic effects with exogenous DHA administration.

## DISCUSSION

Since mammals cannot naturally produce ω-3 fatty acids, they must rely on dietary intake. ω-3 PUFAs found mainly in fish oils or plant oils are required to counteract the more abundant ω-6 fatty acids in the diet. In the current study, we showed that mice engineered to carry a *fat*-1 gene from the roundworm *C. elegans*, adding a double bond into an unsaturated fatty acid hydrocarbon chain and converting ω-6 to ω-3 fatty acids, showed significant protection from chronic colitis-associated carcinogenesis. Further elucidation showed that the intake of more than 3 g ω-3 PUFAs / 60 kg of body weight can cause tissue accumulation of ω-3 PUFAs similar to that found in the colons of *fat-*1 TG mice. ω-3 PUFAs can provide cancer preventive actions in patients with longstanding inflammatory bowel disease, using molecular mechanisms such as blocking β-catenin dissociation complex, inducing tumor suppressive 15-PGDH, inhibiting COX-2 and NF-κB, and inducing selective apoptosis in cancer cells.

Unlike previous reports reaching the similar result of significantly reduced colitis-associated cancer in *fat-*1 TG mice [13, 14], our study is quite novel in revealing the following three cancer preventive mechanisms: blocking of the dissociation of β-catenin to inhibit β-catenin-driven abnormal proliferation, significant induction of tumor suppressive 15-PGDH, and determination of an advisable dietary dose of exogenous ω-3 PUFAs to achieve CAC prevention in clinic. Before our study, based on the concept of the liaison between chronic inflammation, carcinoma and cancer prevention, and the intervention of strong anti-inflammatory agents or nutritional elements, most studies focused on the important role of COX-2-derived PGE_2_, a potent lipid inflammatory mediator, in colitis-associated carcinogenesis [19] and reversal with COX inhibitory agents using non-steroidal anti-inflammatory drugs, 8-hydroxydeoxyguanine, infliximab, curcumin, *etc*. Shown in the current study using ω-3 PUFAs, COX-2 inhibitory action and the associated NF-κB inhibition provided a common mechanism for prevention of both colitis-associated cancer and other types of cancer [11–13, 20, 21]. Over-expression of COX-2 in cultured human colorectal cancer cells enhances PGE_2_ production and promotes tumor growth; conversely depletion of COX-2 attenuates growth [22]. To emphasize why we have presented COX-2 inhibition and NF-κB repression following intake of DHA rather than EPA (Figure 1 & 2), DHA was proven to be superior to both EPA and arachidonic acid for these actions. Considering the facts that individuals with Inflammatory bowel disease (IBD) have a 10- to 40-fold increased risk of developing colorectal cancer compared with those without IBD [23] and that chronic persistent inflamed mucosa progresses through dysplasia to adenocarcinoma, known as the “inflammation-dysplasia-carcinoma sequence” in contrast to the “adenoma-carcinoma sequence” of sporadic colorectal cancer, earlier or continuous intervention with an anti-inflammatory agent targeting the inhibition of COX-2 and redox sensitive NF-κB can be an effective way to reduce or prevent CAC [24].

The initial core cancer preventive mechanisms using ω-3 PUFAs explored in the current CAC model, we found that blocking dissociation of the β-catenin complex was a critical mechanism responsible for anti-tumorigenesis. Previous reports revealed an efficient inhibitory mechanism against proliferation driven by β-catenin activation, a key mediator in WNT regulation of multiple cellular functions in both embryogenesis and tumorigenesis [25]. In the absence of a WNT signal, β-catenin exists within a cytoplasmic complex, a β-catenin destruction complex, along with GSK3β, APC, and Axin, where it is trans-phosphorylated and targeted for degradation by the proteasome, but on activation of WNT signaling, AOM and DSS perturbs this destruction complex, leading to significant cytoplasmic accumulation of β-catenin and allowing its translocation into the cell nucleus. In the nucleus, β-catenin associates with *Tcf/Lef,* which stimulates the transcription of target genes important for proliferation. This blocking action of β-catenin by ω-3 PUFAs has been identified in cancer prevention of cholangiocarcinoma and hepatocellular carcinoma; however, the detailed mechanism for inhibition of β-catenin nuclear translocation and the inhibition of the *Lef* co-activator to block abnormal cellular proliferation by ω-PUFAs is described in the current study.

ω-3 PUFAs seems to be another critical pathway leading to prevention of CAC and CRC. The levels of COX-2 and PGE_2_, the product of COX-2, were significantly increased in both tumor and surrounding non-tumor tissues of colitis-associated cancer. The levels of 15-PGDH, the enzyme responsible for PGE_2_ degradation, were significantly decreased in WT mice, whereas they were significantly preserved in *fat-*1 TG mice (Figure 4D). The preserved and induced expression of 15-PGDH explains the removal of tumorigenic, excess PGE_2_ in colon tissue through metabolic inactivation of PGE_2_ catalyzed by NAD (1)-dependent 15-PGDH. Therefore, 15-PGDH has been speculated to be a physiological antagonist of COX-2 and a tumor suppressor in colon carcinogenesis [26]. 15-PGDH preservation in spite of repressing oncogenic mechanisms can either protect against experimentally induced carcinogenesis or render cancerous or transformed cells susceptible to apoptosis, conclusively counteracting the oncogenic action of PGE_2_ [27], dictating that 15-PGDH is a novel molecular target for cancer chemoprevention and therapy [15].

ω-3 PUFAs can be also a contributing mechanism preventing CAC. In *in vitro* study, Lu *et al.* [9] showed enhanced apoptosis and inhibited GSK-3β phosphorylation to down-regulate both β-catenin and cyclin D1 with *fat*-1 gene transfer directly into prostate cancer cells and in *in vivo* study using a melanoma xenograft model, Xia *et al.* [6] showed that the anti-tumor action of ω-3 PUFAs was mediated through apoptosis via the activation of the Phosphatase and tensin homologue deleted on the chromosome 10 pathway. Apoptosis-inducing action of ω-3 PUFAs can contribute to anti-metastasis actions through down-regulation of cell adhesion-related genes [5]. As seen in the current study, combined with blocking β-catenin activation and cyclin inhibition, ω-3 PUFAs significantly decreased anti-apoptotic genes, survivin, and Bcl-2, and increased apoptotic executors, leading to mitigation of AOM-initiated carcinogenic process. Though Siddiqui *et al.* [28] described synergistic anti-cancer effects of DHA and curcumin against mammary tumors through reduced expression of survivin, we inferred that the inhibitory action of ω-3 PUFAs on survivin/Bcl-2 was a mechanism explored for the first time in the current experiment.

Lastly, though further study is required to show that intake of more than 3 g ω-3 PUFAs/60 kg of body weight can achieve cancer preventive outcomes similar to those seen in *fat-*1 TG mice, findings from *in vitro* and *in vivo* experiments strongly suggest favorable results for ω-3 PUFAs. We presented lipid profiles as the ratio of ω-6 : ω-3 PUFAs, since the reported average ratios of ω-6:ω-3 PUFAs remain 10–4 : 1 in humans other than those with a Western diet and 15–20 : 1 in humans following a Western diet deficient in ω-3 PUFAs [29]; *fat-*1 TG mice showed a ratio of ω-6:ω-3 PUFAs significantly lower than the reported averages (*P* < 0.001) and intake of more than 3 g ω-3 PUFAs / 60 kg of body weight resulted in a ratio similar to that seen in *fat-*1 TG mice (*P* < 0.05). Analysis of the *fat-*1 TG mice group showed an optimal balance of ω-6 : ω-3 PUFAs at 6 : 1, whereas the WT mice group presented ω-6 : ω-3 PUFAs ratio of 17 : 1, but exogenous intake of ω-3 PUFAs such as DHA in the current study, led to an optimal ω-6 : ω-3 PUFA ratio of 8–6 : 1 in colon tissue.

In conclusion, our experimental findings suggested that long-term administration of ω-3 PUFAs as a dietary supplement can be used as a strategy either to prevent CAC in patients with longstanding, recurring, and extensive involvement of IBD or to enable the surveillance of recurrent colon adenoma in patients with endoscopic polypectomy.

## MATERIALS AND METHODS

### Materials and cell culture

DHA among n-3 PUFAs including EPA and AA *etc*, was chosen after a preliminary study that DHA was the most strongly inhibiting activities of cancer cells and purchased from Cayman (Michigan, USA). The HCT116 human colorectal cancer cell line was obtained from ATCC several years ago. The HCT116 human colorectal cancer cell line was cultured in RPMI medium containing 10% (*v*/*v*) fetal bovine serum and 100 U/ml penicillin. Cells were maintained at 37°C in a humidified atmosphere containing 5% CO_2_.

### Antibodies

β-actin, β-catenin, APC, Lamin B, COX-2, phosphorylated IκBα, NFκB p65, NFκB p50, Fas, Bax and survivin antibodies were purchased from Santa Cruz Biotechnology (Santa Cruz, CA). Cyclin D1, Cyclin E, CDK2, CDK4, Caspase-8, Bcl-2, p21, 15-PGDH and GSK3β antibodies were all purchased from Cell Signaling (Denver, MA).

### Cell viability assay

In a cell proliferation assay using 3-(4,5-dimethylthiazol-2-yl)-2,5-diphenyltetra-zolium bromide (MTT; Sigma Aldrich), cells were seeded into 96-well plates at 1–2 × 10^4^ cells/well overnight before drugs were added. The cells were incubated with different concentrations of DHA for 12 h. Cells were incubated with MTT for 2 h and read at an optical density of 570 nm.

### Annexin V for apoptosis analysis

Cells were treated with DHA or phosphate-buffered saline (PBS) as a control. After trypsinization, cells were fixed in 70% ethanol for 30 minutes at 4°C. Apoptosis was analyzed by fluorescence-activated cell sorting using the Annexin V-FITC Apoptosis Detection kit (BD Biosciences, CA). Flow cytometric analysis was completed and the proportion of cells was assessed by the histograms generated using the computer program Mod-Fit.

### Enzyme-linked immunosorbent assay (ELISA)

Cells were homogenized with 1 ml of cell lysis buffer (Cell Signaling, Denver, MA) containing 1 mM of PMSF, incubated for 20 min, and centrifuged at 10,000 × *g* for 10 min. Supernatants were re-centrifuged and collected. All samples were stored at −80°C until required. Mouse PGE_2_ (R&D Systems, Minneapolis, MN) ELISAs were performed according to manufacturer instructions.

### Luciferase reporter activity assay

Cultured cells were seeded at a concentration achieving 80% confluence in 12-well plates for 24 h prior to transfection. The cells were transiently transfected with 0.2 μg/well of a translucent *Tcf*/*Lef*-*Luc* reporter vector, which was designed to measure the transcriptional activity of *Tcf*/*Lef*-responsive genes. After transfection, the cells were treated with DHA in serum-free medium at the indicated time periods. The cell lysates were then obtained with 1 × reporter lysis buffer (Promega, Madison, WI). The luciferase activity was assayed using a Victor^2^ luminometer (Perkin Elmer, Waltham, MA). The relative luciferase activity was calculated after normalization of cellular proteins. All values are expressed as the percentage of activity relative to basal activity.

### Immunohistochemical staining

After paraffin blocks were dewaxed and rehydrated with graded alcohol, tissue sections were heated in pressure jars filled with 10 mM/L citrate buffer in a microwave for 10 minutes. Slides were cooled in water for 15 min and washed in PBS. The slides were incubated overnight with the primary antibody. After incubation, a subsequent reaction was formed using a VECTOR kit (Vector Laboratories, Inc., Burlingame, CA). Finally, the slides were incubated with 3, 3′-diaminobenzidine (Invitrogen Life Technologies, Carlsbad, CA) and counterstained with hematoxylin (Sigma Aldrich, St. Louis, MO).

### TUNEL staining

Apoptosis was visualized using a terminal deoxynucleotidyl transferase (TdT) fRAGel DNA fragmentation Detection kit (Oncogene Research Products, La Jolla, CA). After routine deparaffinization, rehydration, and washing in PBS (pH 7.4), tissues were digested with proteinase K (20 μg/mL in PBS) for 20 min at room temperature and washed. Tissues were incubated in equilibration buffer for 10 min and treated with TdT enzyme at 37°C for 1 h. To determine the apoptotic index in each group, TUNEL-immunostained sections were scanned under low-power magnification (× 100) to locate the apoptotic hotspots.

### Animals and study protocol

The C57BL/6 mice were purchased from Orient bio (Seoul, Korea) and *fat-1* TG mice on a pure C57BL/6 background were donated by Dr. Kang JX (Harvard Medical School, Boston, MA), respectively. They were bred and genotyped in our facility for the project. The *fat-1* TG mice were verified as previously described by PCR using tail DNA extracted. The sequences of PCR primers were recommended by Jackson Laboratories and Dr. Kang JX. As shown in Figure 3A, A total of 40 mice were divided into four groups, 10 mice per group, respectively; Group1 (WT C57BL/6 background) and Group 3 (*fat-1* TG) were injected with PBS *i.p.* and drank tap water, Group 2 (C57BL/6 wild type) and Group 4 (*fat-1* TG) were given AOM 5 mg/kg injection followed by 1 cycle of 2.5% DSS ingestion for 1 week after which mice were drank tap water (Figure 3A). Mice were fed sterilized commercial pellet diets and sterile water *ad libitum* and housed in an air-conditioned biohazard room at a temperature of 24°C. Animals were handled in an accredited animal facility in accordance with international policies of animal center of the CHA University. After 16 weeks all mice were killed and colons were removed, opened longitudinally, and rinsed with PBS. The number and size of gross polypoid tumors were charted. Isolated tissues from distal one-third of colon were subjected to a histologic examination and mucosal scratches of tissues from proximal two-third part were subjected to immediate freezing for the extraction of protein.

### Statistical analysis

All the experiments in this study except *in vivo* animal experiment were repeated more than thrice and the results are expressed as the mean ± standard deviation. The data were analyzed by one-way analysis of variance, and the statistical significance between groups was determined by Duncan's multiple range test. Statistical significance was accepted at *P* < 0.05.

## References

[1] Kang JX, Wang J, Wu L, Kang ZB (2004). Transgenic mice: fat-1 mice convert n-6 to n-3 fatty acids. Nature.

[2] Guo T, Liu XF, Ding XB, Yang FF, Nie YW, An YJ, Guo H (2011). Fat-1 transgenic cattle as a model to study the function of omega-3 fatty acids. Lipids Health Dis.

[3] Spychalla JP, Kinney AJ, Browse J (1997). Identification of an animal omega-3 fatty acid desaturase by heterologous expression in Arabidopsis. Proc Natl Acad Sci USA.

[4] Kang JX (2007). Fat-1 transgenic mice: a new model for omega-3 research. Prostaglandins Leukot Essent Fatty Acids.

[5] Xia SH, Wang J, Kang JX (2005). Decreased n-6/n-3 fatty acid ratio reduces the invasive potential of human lung cancer cells by downregulation of cell adhesion/invasion-related genes. Carcinogenesis.

[6] Xia S, Lu Y, Wang J, He C, Hong S, Serhan CN, Kang JX (2006). Melanoma growth is reduced in fat-1 transgenic mice: impact of omega-6/omega-3 essential fatty acids. Proc Natl Acad Sci USA.

[7] Mohammed A, Janakiram NB, Brewer M, Duff A, Lightfoot S, Brush RS, Anderson RE, Rao CV (2012). Endogenous n-3 polyunsaturated fatty acids delay progression of pancreatic ductal adenocarcinoma in Fat-1-p48(Cre/+)-LSL-Kras(G12D/+) mice. Neoplasia.

[8] Zou Z, Bellenger S, Massey KA, Nicolaou A, Geissler A, Bidu C, Bonnotte B, Pierre AS, Minville-Walz M, Rialland M, Seubert J, Kang JX, Lagrost L (2013). Inhibition of the HER2 pathway by n-3 polyunsaturated fatty acids prevents breast cancer in fat-1 transgenic mice. J Lipid Res.

[9] Lu Y, Nie D, Witt WT, Chen Q, Shen M, Xie H, Lai L, Dai Y, Zhang J (2008). Expression of the fat-1 gene diminishes prostate cancer growth *in vivo* through enhancing apoptosis and inhibiting GSK-3 beta phosphorylation. Mol Cancer Ther.

[10] Weylandt KH, Krause LF, Gomolka B, Chiu CY, Bilal S, Nadolny A, Waechter SF, Fischer A, Rothe M, Kang JX (2011). Suppressed liver tumorigenesis in fat-1 mice with elevated omega-3 fatty acids is associated with increased omega-3 derived lipid mediators and reduced TNF-alpha. Carcinogenesis.

[11] Lim K, Han C, Dai Y, Shen M, Wu T (2009). Omega-3 polyunsaturated fatty acids inhibit hepatocellular carcinoma cell growth through blocking beta-catenin and cyclooxygenase-2. Mol Cancer Ther.

[12] Algamas-Dimantov A, Yehuda-Shnaidman E, Hertz R, Peri I, Bar-Tana J, Schwartz B (2014). Prevention of diabetes-promoted colorectal cancer by (n-3) polyunsaturated fatty acids and (n-3) PUFA mimetic. Oncotarget.

[13] Nowak J, Weylandt KH, Habbel P, Wang J, Dignass A, Glickman JN, Kang JX (2007). Colitis-associated colon tumorigenesis is suppressed in transgenic mice rich in endogenous n-3 fatty acids. Carcinogenesis.

[14] Jia Q, Lupton JR, Smith R, Weeks BR, Callaway E, Davidson LA, Kim W, Fan YY, Yang P, Newman RA, Kang JX, McMurray DN, Chapkin RS (2008). Reduced colitis-associated colon cancer in Fat-1 (n-3 fatty acid desaturase) transgenic mice. Cancer Res.

[15] Han YM, Park JM, Cha JY, Jeong M, Go EJ, Hahm KB (2016). Endogenous conversion of omega-6 to omega-3 polyunsaturated fatty acids in fat-1 mice attenuated intestinal polyposis by either inhibiting COX-2/beta-catenin signaling or activating 15-PGDH/IL-18. Int J Cancer.

[16] De Robertis M, Massi E, Poeta ML, Carotti S, Morini S, Cecchetelli L, Signori E, Fazio VM (2011). The AOM/DSS murine model for the study of colon carcinogenesis: From pathways to diagnosis and therapy studies. J Carcinog.

[17] Ahmed AU, Williams BR, Hannigan GE (2015). Transcriptional Activation of Inflammatory Genes: Mechanistic Insight into Selectivity and Diversity. Biomolecules.

[18] Simopoulos AP (2008). The importance of the omega-6/omega-3 fatty acid ratio in cardiovascular disease and other chronic diseases. Exp Biol Med (Maywood).

[19] Nakanishi M, Rosenberg DW (2013). Multifaceted roles of PGE2 in inflammation and cancer. Semin Immunopathol.

[20] Gravaghi C, La Perle KM, Ogrodwski P, Kang JX, Quimby F, Lipkin M, Lamprecht SA (2011). Cox-2 expression, PGE and cytokines production are inhibited by endogenously synthesized n-3 PUFAs in inflamed colon of fat-1 mice. J Nutr Biochem.

[21] Camuesco D, Galvez J, Nieto A, Comalada M, Rodriguez-Cabezas ME, Concha A, Xaus J, Zarzuelo A (2005). Dietary olive oil supplemented with fish oil, rich in EPA, DHA (n-3) polyunsaturated fatty acids, attenuates colonic inflammation in rats with DSS-induced colitis. J Nutr.

[22] Han C, Wu T (2015). Cyclooxygenase-2-derived prostaglandin E2 promotes human cholangiocarcinoma cell growth and invasion through EP1 receptor-mediated activation of the epidermal growth factor receptor and Akt. J Biol Chem.

[23] Rosario F, Michela C, Elisa C, Caristo G, Fornaro F, Giovinazzo D, Sticchi C, Casaccia M, Andorno E (2016). Colorectal Cancer in Patients With Inflammatory Bowel Disease: The Need for a Real Surveillance Program. Clin Colorectal Cancer.

[24] Kwak MK, Kensler TW (2010). Targeting NRF2 signaling for cancer chemoprevention. Toxicol Appl Pharmacol.

[25] Madan B, Walker MP, Young R, Quick L, Orgel KA, Ryan M, Gupta P, Henrich IC, Ferrer M, Marine S, Roberts BS, Arthur WT, Berndt JD (2016). USP6 oncogene promotes Wnt signaling by deubiquitylating Frizzleds. Proc Natl Acad Sci USA.

[26] Na HK, Park JM, Lee HG, Lee HN, Myung SJ, Surh YJ (2011). 15-Hydroxyprostaglandin dehydrogenase as a novel molecular target for cancer chemoprevention and therapy. Biochem Pharmacol.

[27] Tai HH (2011). Prostaglandin catabolic enzymes as tumor suppressors. Cancer Metastasis Rev.

[28] Siddiqui RA, Harvey KA, Walker C, Altenburg J, Xu Z, Terry C, Camarillo I, Jones-Hall Y, Mariash C (2013). Characterization of synergistic anti-cancer effects of docosahexaenoic acid and curcumin on DMBA-induced mammary tumorigenesis in mice. BMC cancer.

[29] Allen IC, TeKippe EM, Woodford RM, Uronis JM, Holl EK, Rogers AB, Herfarth HH, Jobin C, Ting JP (2010). The NLRP3 inflammasome functions as a negative regulator of tumorigenesis during colitis-associated cancer. J Exp Med.

